# Nerve Hydrodissection as Treatment for Entrapment Neuropathies: A Literature Review

**DOI:** 10.7759/cureus.101039

**Published:** 2026-01-07

**Authors:** Szymon Stupnicki, Jakub Tarczykowski, Aleksandra Oparcik, Mikołaj Zakryś, Katarzyna A Zakryś, Mateusz Szot, Natalia Kwaśniewska

**Affiliations:** 1 General Practice, Wojewódzki Szpital Wielospecjalistyczny im. Dr. Jana Jonstona w Lesznie, Leszno, POL; 2 General Practice, Uniwersytecki Szpital Kliniczny w Poznaniu, Poznań, POL; 3 General Practice, Centrum Medyczne HCP, Poznań, POL; 4 General Practice, Szpital Wojewódzki w Poznaniu, Poznań, POL; 5 General Practice, Wojskowy Instytut Medyczny - Państwowy Instytut Badawczy, Warszawa, POL

**Keywords:** hydrodissection, nerve regeneration, perineural injection therapy, peripheral entrapment neuropathy, ultrasound-guided

## Abstract

Entrapment neuropathies are disorders in which peripheral nerves are affected at anatomically restricted sites or at locations where they are vulnerable to external compression. They are prevalent in the general population and may lead to pain, sensory disturbances, and functional impairment. In recent years, nerve hydrodissection has gained attention as a minimally invasive, ultrasound-guided treatment option for entrapment neuropathies. This technique involves the injection of different substances into the perineural space to mechanically separate the nerve from adjacent tissues and potentially reduce compression. The aim of this narrative review was to summarize recent evidence regarding the clinical utility of nerve hydrodissection in the management of entrapment neuropathies in different anatomical regions. The available literature indicates that nerve hydrodissection is associated with meaningful improvements in pain and functional outcomes across a variety of entrapment neuropathies, with the largest body of evidence related to carpal tunnel syndrome. Overall, 5% dextrose in water has emerged as a commonly used injectate with a favorable clinical profile. Nerve hydrodissection appears to be a promising addition to conservative treatment strategies for entrapment neuropathies, particularly in mild to moderate cases. Future studies should focus on entrapment neuropathies other than carpal tunnel syndrome to provide higher-quality and more comprehensive evidence in these conditions.

## Introduction and background

Entrapment neuropathies are conditions in which a nerve becomes damaged due to compression by the surrounding local tissues. This most commonly occurs in anatomical regions where available space for the nerve is limited. A typical example is carpal tunnel syndrome (CTS), the most prevalent entrapment neuropathy worldwide. In conditions such as CTS, inflammatory changes associated with repetitive overuse may increase pressure within the carpal tunnel, while systemic disorders, including autoimmune diseases or diabetes, may further contribute to nerve dysfunction [1,2]. Another cause of entrapment neuropathy is the superficial location of the nerve, which makes it susceptible to chronic external compression. This is observed, for instance, in neuropathies of the lateral femoral cutaneous nerve or the common peroneal nerve [3,4]. It is important to highlight that a large proportion of entrapment neuropathies remains idiopathic [2].

The prevalence of entrapment neuropathies in the general population is relatively high. It is estimated that CTS affects between 5% and 16% of the population, depending on the region [1]. The second most common mononeuropathy in the upper limb, cubital tunnel syndrome, affects around 5.9% of the population in the St. Louis metropolitan area in the United States [5]. The overall prevalence of all entrapment neuropathies is difficult to determine. However, statistics about the two most common conditions discussed above illustrate their significant commonness. Appropriate therapeutic decisions are made taking into account different factors: the severity and progression of symptoms, duration of complaints, nerve conduction study findings, ultrasound imaging, and patient demands. Treatment may be conservative or surgical [6].

In recent years, numerous studies have examined a novel conservative treatment option for entrapment neuropathies: nerve hydrodissection (HD), also referred to as perineural injection therapy (PIT). This technique involves injecting various substances around the nerve. The procedure is performed under ultrasound guidance using a standard needle attached to a syringe. Substances used in research protocols include 5% dextrose in water (DW), local anesthetics (LAs), hyaluronic acid (HA), platelet-rich plasma (PRP), corticosteroids (CS), normal saline (NS), and hyaluronidase [7]. Perineural injections may provide two types of beneficial effects: a pharmacological effect, stemming from the inherent action of the injected agent near the nerve, and a mechanical effect, resulting from releasing the nerve from surrounding adhesions that may be contributing to entrapment [7,8].

Lately, an increasing number of studies have investigated nerve HD as a potential conservative treatment for entrapment neuropathies. The expanding body of literature on this technique over the past five years provided the rationale for conducting the present narrative review.

Methodology

This study is a narrative review of the recent literature on the effectiveness of HD in the treatment of entrapment neuropathies of different anatomical locations. The PubMed database was searched in September 2025. Peer-reviewed articles in English published from 2020 onward were included in this review. Studies that did not refer to neuropathies were excluded. We focused on systematic reviews and RCTs, as they represent the highest level of evidence. However, studies with lower hierarchy were also included, especially when we reported on less common entrapment neuropathies with lower incidence. As this is a narrative review, no new statistical analyses were performed. All statistical data and quantitative outcomes presented are based exclusively on the results reported in the included primary studies and available meta-analyses.

## Review

Carpal tunnel syndrome

It is appropriate to begin with the first recently published meta-analysis on the topic of HD, addressing the effectiveness of various injectates used in the treatment of CTS. Lee et al., in their valuable study, analyzed nine RCTs including a total of 458 patients, in which both the experimental and control groups received different injectable agents [9]. The analyzed substances included NS, 5% DW, CS, PRP, and hyaluronidase. Based on the studies included in their meta-analysis, the primary outcome measure was the Boston Carpal Tunnel Questionnaire (BCTQ), comprising the Symptom Severity Scale (SSS) and the Functional Status Scale (FSS). The effectiveness of treatment by each substance was evaluated using the surface under the cumulative ranking curve (SUCRA). The authors found that for BCTQ symptom severity, DW demonstrated the highest SUCRA value (99.9 at week 4), whereas PRP achieved the highest SUCRA values at weeks 12 and 24 (95.7 and 93.9, respectively). Regarding BCTQ functional outcomes, DW was ranked as the most effective intervention, with SUCRA values of 99.9, 89.8, and 88.8 at weeks 4, 12, and 24, respectively.

These findings are consistent with previously published systematic reviews, where DW and PRP appeared to be the most effective interventions for improving VAS and BCTQ scores. Compared to CS, DW and PRP have longer-lasting therapeutic effects [10,11]. Retrospective data further support these observations, demonstrating favorable short- and mid-term outcomes (after one month and six months) for DW and PRP compared with NS or HA [12]. With regard to HA, which did not appear in the large meta-analysis and aforementioned systematic reviews, Su et al., in their RCTs, investigated the effectiveness of this substance in mild to moderate CTS [13]. A total of 35 patients were evaluated: 18 received 2.5 mL HA and 17 received 2.5 mL NS. Outcomes were measured prior to injection and at two weeks, and at one, three, and six months. It was found that a single HA injection did not show significantly superior outcomes compared to NS, except for BCTQ and Numerical Rating Scale (NRS) at the second week post-injection. Both groups demonstrated statistically significant improvement as early as two weeks, with the best clinically significant results observed six months after injection. This contrasts with the results presented by Elawamy et al., who used a substance that acts antagonistically to HA: hyaluronidase [14]. In a total group of 60 patients, they showed that HD using 10 mL NS + 1500 units of hyaluronidase resulted in clinically and statistically significant improvement in BCTQ (both FSS and SSS) and VAS compared to the group that received only 10 mL NS. These significant differences already appeared one week after injection and persisted until the final six-month follow-up.

Attention should also be directed to the volume of the injectate. Lee et al. in their meta-analysis concluded that the use of 5 mL was more effective compared with other volumes (1, 2, 4, and 10 mL) [9]. However, the studies included in their meta-analysis did not incorporate many trials using a 10 mL injectate volume. In addition, a study by Eyvaz et al. [15], published later, was not included. In this work, the authors compared the use of four types of injectates: 5 mL NS, 10 mL NS, 5 mL DW, and 10 mL DW, in a group of 80 patients with CTS. Statistically significant improvements in BCTQ and VAS scores were observed across all groups at the final three-month follow-up. However, the 10 mL DW group showed considerably greater improvements in BCTQ scores compared to the 5 mL NS group. In the case of visual analog scale (VAS) scores, 10 mL DW was statistically superior to both NS groups. For the 5 mL DW group, no statistically significant differences were demonstrated for either VAS or BCTQ compared with the other groups [15]. The study by Lin et al. is also consistent with these findings, proving that increasing the volume of injectate is favourable [16]. They demonstrated in a group of 63 wrists that the largest volume, 4 mL DW, was more effective than 2 mL DW or 1 mL DW. However, they did not observe statistically significant differences in VAS or BCTQ between the groups at the final 24-month follow-up. Based on these studies, we can conclude that a volume of 4-10 mL is optimal for achieving the best HD effect using DW. The upper end of this range may potentially be more beneficial, though this requires verification in further studies.

Finally, it is worth adding a few words about the HD technique itself in CTS. HD can be performed in two ways: one called the short-axis or in-plane technique, and the other called the long-axis or out-of-plane technique. The names of these techniques refer to the relationship between the needle orientation and the axis of the nerve [7]. Chen et al., in a group of 44 patients (21 wrists treated with the short-axis technique and 23 wrists treated with the long-axis technique) using 5 mL NS, concluded that both techniques are similarly effective in mild to moderate CTS. BCTQ at 6-month follow-up did not differ statistically [17]. In general, the short-axis technique is considered easier to learn and less technically demanding. Therefore, it should be recommended for clinicians beginning to perform HD, especially since, according to available data, it does not yield worse outcomes [7]. Furthermore, HD is considered to be a safe intervention in CTS. Sveva et al., in their systematic review, based on 15 studies comprising a total of 923 patients, identified that procedural side effects were observed in only 11 cases [18].

An analysis of these studies shows that regardless of the injectate volume and type of substance used, HD resulted in statistically and clinically significant improvement from baseline in VAS and BCTQ. However, platelet-rich plasma and 5% DW emerge as the most extensively studied injectates with the strongest clinical efficacy. It is difficult to provide a definitive explanation as to why DW proved to be the most effective intervention in terms of functional improvement. However, this agent resulted in the greatest improvement in sensory nerve conduction velocity [9,11]. A hallmark of CTS is the loss of manual dexterity due to associated impairment of sensation and proprioception [19,20]. It may therefore be hypothesized that improvement in sensory conduction facilitated by DW uniquely contributed to the restoration of proprioception and sensation and, consequently, functional capacity. PRP, on the other hand, is well known for its extensively studied regenerative potential, attributed to its high concentration of various growth factors. It is well established that PRP promotes the regeneration of various tissues through modulation of the inflammatory response, which consists of several distinct phases. Tissue repair and remodeling occur only in the later stages of this cascade [21]. This may explain why PRP was the most effective intervention in symptom severity over a longer follow-up period (12 and 24 weeks), while it did not demonstrate superior efficacy in the short-term follow-up (four weeks) [9]. It is also worth noting that HA is a glycosaminoglycan, a polysaccharide capable of binding large amounts of water [22]. This effect is beneficial in degenerative joint diseases, where HA acts as a lubricant and improves symptoms [23]. However, in CTS, we are dealing with increased pressure in the carpal tunnel, and introducing a substance that retains water does not seem to be a good idea [24]. Hyaluronidase, in contrast, causes the breakdown of hyaluronic acid, resulting in reduced tissue hydration. These facts may explain why this particular substance produced better results than NS [25].

Ulnar nerve: cubital tunnel

Regarding ulnar nerve neuropathy, substantially fewer data are available than for CTS. A simple explanation may be that this condition is far less common [5,26]. Following the line of evidence from CTS studies, Chen et al. decided in their double-blind RCTs to investigate whether, in ulnar neuropathy at the elbow (UNE), DW would also yield better outcomes than CS [27]. Their study was conducted on 33 patients, of whom 17 received 5 mL DW and 16 received 3 mL CS consisting of 10 mg/mL triamcinolone acetonide mixed with 2 mL NS. They did not demonstrate statistically significant differences between the groups in the VAS scores, which they selected as the primary outcome (at the one-, three-, four-, and six-month follow-ups). However, the DW group showed better results beginning from the third month onward. Moreover, from month three in the CS group, Chen et al. observed a tendency toward recurrence of pain symptoms, which did not occur in the DW group [27]. On the other hand, Wu et al., in a group of 54 wrists, showed that in CTS, compared with the steroid group, the DW group exhibited a clinically and statistically significant reduction in pain and disability through the fourth to the sixth month [28]. It is possible that Chen et al. were unable to demonstrate statistically significant differences in favor of DW due to the small sample size or insufficiently long follow-up. Another possibility is that these different findings result from the distinct pathophysiology and anatomy of the carpal tunnel versus the ulnar nerve canal.

A subsequent study by Hooper et al., based on a case series of three UNE cases, also demonstrated the effectiveness of HD [29]. For the perineural injection procedure, they used platelet lysate combined with 50% dextrose and 0.5% ropivacaine to create a 5% DW neuroprolotherapy solution. They reported improvement in patients with baseline VAS scores of 6/10, 6/10, and 3/10, which improved to 0/10, 0/10, and 1/10 at final follow-up (18, 22, and 67 months after HD, respectively). Gerard et al. reported what they believe to be the first case of peripheral nerve HD of the ulnar nerve in an overhead throwing athlete [30]. In their case, a baseball pitcher presented with a one-week history of medial elbow pain and paresthesia in the fourth and fifth digits occurring only during end-range throwing, without trauma, and with no symptoms at rest. HD was performed with a solution of 2 mL 1% lidocaine, 7 mL normal saline, and 1 mL of 6 mg/mL betamethasone. Four days after the procedure, he pitched a full game at his expected level. At seven months post procedure, the athlete continued collegiate pitching without pain or limitations.

Meralgia paresthetica (MP): lateral femoral cutaneous nerve entrapment

A substantial amount of new data on HD in MP was provided by Shi et al.'s study in 2024, in which they compared ultrasound-guided HD using DW or CS injections for treating MP [31]. Fifty-six patients received a single perineural injection of either 10 mL DW or a CS mixture. Both treatments significantly reduced VAS pain scores and improved VAS global quality-of-life ratings, but the DW group showed greater symptom relief and significantly higher clinical success at four to six months post treatment. At the final six-month follow-up, 85.7% (24/28) of patients in the DW group achieved a successful clinical response vs. 50.0% (14/28) in the CS group. Additionally, no adverse effects occurred in the DW group, whereas six patients in the CS group reported side effects. To our knowledge, this is the first study conducted on such a large group of patients, and additionally, the first RCT on HD in MP. These results indicate that DW HD may offer a safer and more effective long-term treatment option for MP than CS [31]. These findings are supported by the work of Su et al., who described a case of a 35-year-old woman with chronic MP lasting an exceptionally long time: 20 years [32]. Conventional treatments were ineffective, so ultrasound-guided nerve HDs with 10 mL DW were performed. After seven sessions over two months, their patient showed significant symptomatic and electrophysiological improvement. Ultrasound imaging also demonstrated a reduction in nerve swelling. Their report suggests that in chronic, long-standing MP, multiple therapeutic injections may be more effective than a single dose. Another case report involving a 46-year-old male with six-year MP symptoms described successful treatment with a single injection of 20 mL of 1% lignocaine with particulate methylprednisolone 20 mg [33]. The NRS score before the procedure was 5/10. On day 15 after the procedure, the NRS score was 1/10, on day 30, 1/10, at three months, 1/10, and at six months, 2/10. The authors, Tople et al., noted that following HD, the patient was able to return to daily activities.

Based on these findings, we can conclude that HD in MP is an effective treatment method. It may be offered to patients instead of neurotropic medications such as gabapentin, pregabalin, or amitriptyline, which frequently cause adverse effects including somnolence, dizziness, and nausea [34]. However, it must be noted that the number of reports on treating this condition with HD is still limited, and further confirmation of its effectiveness in larger populations would be beneficial.

Sciatic nerve

Several recent studies have examined the use of HD in sciatic nerve neuropathy. Yen et al., in their case series, used HD with a 10 mL mixture containing distilled water, 0.2% lidocaine, and 4 mg of betamethasone in 53 patients with deep gluteal syndrome, a condition involving sciatic nerve entrapment. The primary outcome was the NRS pain score. The NRS score before the procedure was 6.4 ± 1.6. This score significantly decreased to 3.1 ± 1.8, 3.0 ± 1.8, 3.1 ± 2.0, and 3.0 ± 2.1 at one week, one month, three months, and at the final follow-up, respectively. In contrast, at the final follow-up, only 62.3% of the patients maintained a favorable outcome defined as >50% pain reduction. However, the authors did not precisely report the proportions of the mixture used during HD. They also did not objectively evaluate functional outcomes, focusing solely on pain. Additionally, this was a single-arm study without a control group, which limits its value [35].

Yoon et al. reported the case of a 51-year-old woman with progressive, severe left-sided sciatica persisting for several months [36]. Plain radiography and musculoskeletal ultrasound revealed calcification of the left sacrospinous ligament and enlargement of the sciatic nerve. The patient underwent three ultrasound-guided HD sessions using 4 mL of 10% dextrose in water combined with 1 mL of 2% lidocaine (without epinephrine) at the site of calcification, followed by a structured rehabilitation program. Within one month, pain during sitting and walking markedly decreased, with the VAS dropping from 10 to 2. All previously positive provocation tests became negative, and full functional recovery was achieved. However, it remains unclear to what extent the strict rehabilitation regimen contributed to the therapeutic success, as it may have substantially influenced symptom improvement [36]. Park et al. described a rare case of sciatic neuropathy caused by piriformis muscle rhabdomyolysis in a 27-year-old man after strenuous exercise [37]. After initial intensive hydration focused on treating rhabdomyolysis, the VAS score for left buttock pain radiating to the sole was 7, and the patient was unable to walk independently. Pelvic MRI showed edematous changes in the soft tissues surrounding the left sciatic nerve. They decided to perform HD of the sciatic nerve using 5 mL of 1 mg dexamethasone and 5 mL of distilled water. Two weeks post injection, the VAS score decreased to 5, and the patient was able to walk indoors independently. Five months after the injection, the VAS score was 1. The main limitation of this case report is that it is unclear whether symptom resolution resulted from rest or from the HD itself over time [37].

Summary of findings

The HD technique has been most thoroughly investigated in CTS, and its clinical utility is supported by a large amount of evidence. In other entrapment neuropathies, although preliminary findings are similarly encouraging, data remain limited and warrant confirmation through larger, well-designed randomized controlled trials. Furthermore, there is a lack of placebo-controlled studies. NS cannot be treated as a placebo agent because it may have a potential decompressive (mechanical) effect, which is the very essence of HD [38]. Most of the studies included in this review assessed the effectiveness of various injectates in mild or moderate CTS. It is logical that in advanced neuropathies, manifesting as muscle atrophy or complete loss of sensory conduction on electrodiagnostic testing, surgical decompression would likely represent a more effective treatment approach [6,39].

HD represents an effective modality in the treatment of entrapment neuropathies, enabling clinically significant improvement in both function and symptoms across neuropathies of various anatomical locations. The magnitude of clinical benefit depends on the type of solution used during HD. However, regardless of the type of injectate used, the vast majority of studies demonstrate a significant improvement from baseline symptoms. Currently, the most promising and extensively studied injectate is DW. It demonstrates favorable efficacy and a longer therapeutic effect compared with CS and LAs. Moreover, unlike CS, DW is not associated with steroid-related adverse effects, which may include skin hypopigmentation and atrophy, local fat loss, or local infection [40]. Additionally, HD with DW is associated with lower procedural costs relative to PRP, which requires specialized equipment [41]. CS also has a low and unfavorable cost-effectiveness in neuropathies [42]. Therefore, compared with PRP and CS, DW is the most cost-effective option, although this has not been clearly confirmed in dedicated studies. Besides these major strengths of DW, there is no direct knowledge about its pharmacological mechanism of action. It has been hypothesized that such injectates may induce downregulation of the transient receptor potential vanilloid 1 (TRPV1) receptor, whose upregulation has been documented in neuropathic pain states. However, this proposed mechanism has not yet been directly demonstrated in experimental or clinical studies [43,44]. HA is suboptimal due to its mechanism of action and its overall modest performance in published studies, although it should be verified in other entrapment neuropathies other than CTS. 

In the current situation, it appears that the wider use of HD in the treatment of entrapment neuropathies may provide clinically significant benefits and improve the course of therapy. Standard treatment of these conditions using conservative techniques (patient education, rest, splinting, activity modification, NSAIDs) is characterized by limited effectiveness. In CTS, 57-66% of patients require surgical treatment after unsuccessful conservative therapy within up to three years of its initiation [45]. In cubital tunnel syndrome, only 44-66% of patients experience resolution of symptoms within one year after conservative treatment [46]. Surgical interventions are also not free from recurrences and are associated with perioperative complications [47]. Therefore, HD has the potential to contribute to increased overall therapeutic success in the coming years by improving the overall effectiveness of treatment for these conditions.

It is important to acknowledge the main limitation of our study, which stems from its design. As this is a narrative review, it does not employ the rigorous methodology characteristic of a systematic review. Consequently, a certain degree of subjectivity in the selection and interpretation of the literature cannot be excluded. Moreover, no new statistical analyses were performed in the context of this study. Additionally, our review also includes studies with a low level of scientific hierarchy. We selected them because, despite the lower level of evidence, our intention was to demonstrate that HD is reported across other conditions beyond CTS with favourable outcomes.

## Conclusions

HD is an effective therapeutic modality for entrapment neuropathies, with the strongest and most consistent evidence supporting its use in CTS. Although preliminary results in other entrapment neuropathies are encouraging, the current body of evidence remains limited and underscores the need for larger, high-quality randomized controlled and placebo-controlled trials. Among the available injectates, 5% DW appears to offer the most favorable balance of efficacy, safety, duration of effect, and cost-effectiveness, although its precise mechanism of action remains incompletely understood. HD may represent a valuable alternative or adjunct to conventional conservative management, potentially reducing the need for surgical intervention, particularly in mild to moderate disease.
